# Supplementary material for: A human lung alveolus-on-a-chip model of acute radiation-induced lung injury
Source: Nat Commun. 2023 Oct 16;14:6506. doi: 10.1038/s41467-023-42171-z (PMC10579267; doi:10.1038/s41467-023-42171-z)
Supplement: Supplementary file 1 — Supplementary Information [file 41467_2023_42171_MOESM1_ESM.pdf]

SUPPLEMENTARY INFORMATION

A human lung alveolus-on-a-chip model of acute radiation-induced lung injury

Queeny Dasgupta[1 2], Amanda Jiang[1 2], Amy M. Wen[2], Robert Mannix[1], Yuncheng Man [1 2], Sean Hall[2], Emilia Javorsky[2], Donald E. Ingber[1 2 3]

[1] Vascular Biology Program and Department of Surgery, Boston Children's Hospital and Harvard Medical School, Boston, MA 02115, USA

[2] Wyss Institute for Biologically Inspired Engineering at Harvard University, Boston, MA 02115, USA

[3] Harvard John A. Paulson School of Engineering and Applied Sciences, Harvard University, Cambridge, MA 02139, USA

Address all correspondence to: Donald Ingber, MD,PhD (em: don.ingber@wyss.harvard.edu)

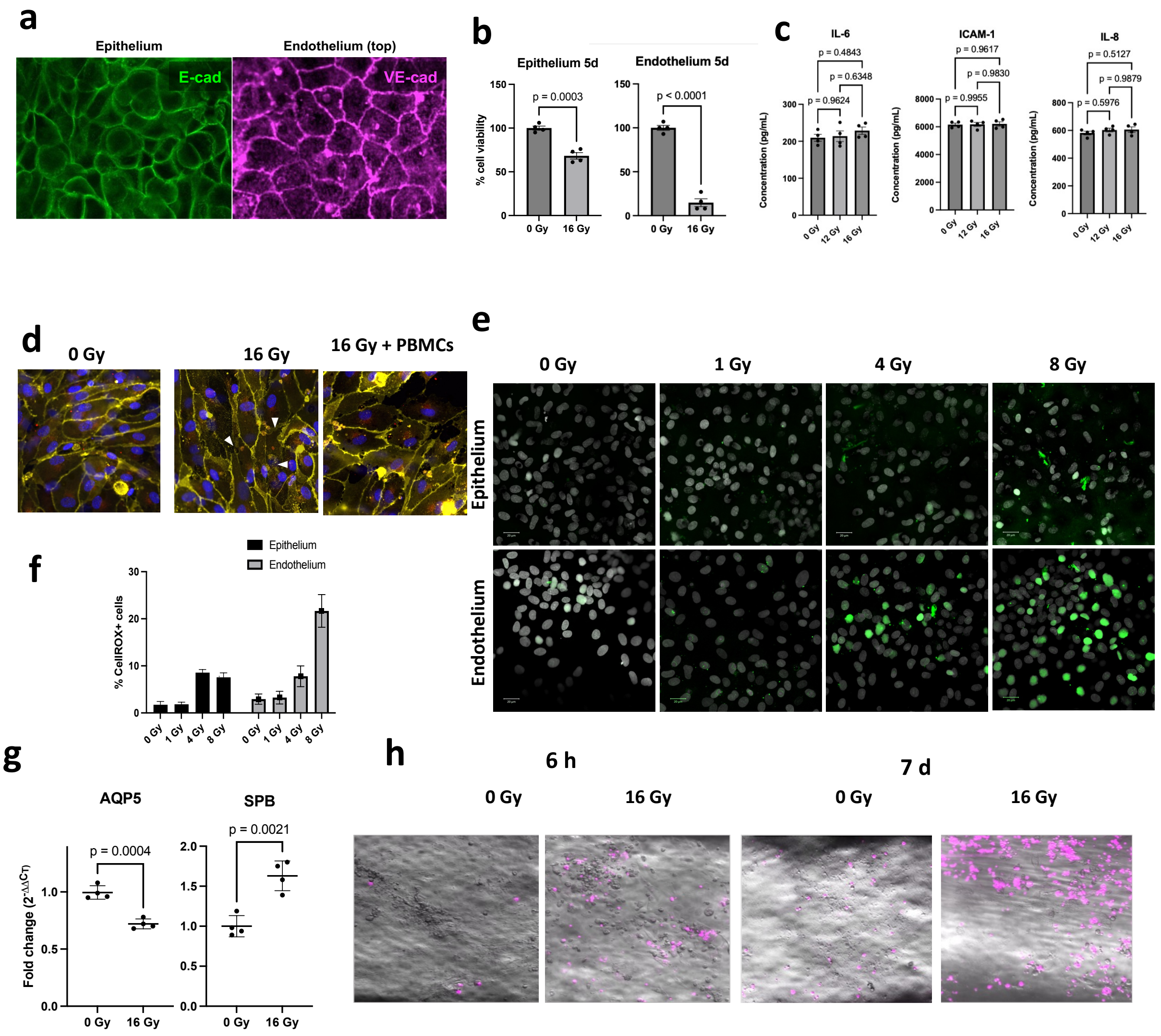

Supplementary Figure S1: (a) Confocal images of epithelial cells stained for E-cadherin(green) and endothelial cells stained for VE-cadherin adherens junction protein (magenta). Scale bar = 20 µm. (b) Cellular viability 5 d after radiation exposure evaluated by Alamar Blue assay. (c) Representative comparison of cytokine levels, 24 h post-radiation showed that 12 Gy and16 Gy radiation did not show an elevation in cytokine levels, in the absence of PBMCs (d) Presence of PBMCs in the endothelial compartment during radiation aggravates tight junction disruption. (e) Dose-dependent ROS expression in the epithelium and

endothelium, shown by CellROX green+ cells and (f) associated quantification (g). Changes in expression of gene markers of Type 1 (aquaporin 5; AQP5) and 2 (surfactant protein B; SPB) alveolar epithelial cells 6 h after exposure to 16 Gy radiation (h). Fluorescence microscopic images showing PBMC recruitment to the endothelial side at 6 h and 7d post-radiation.

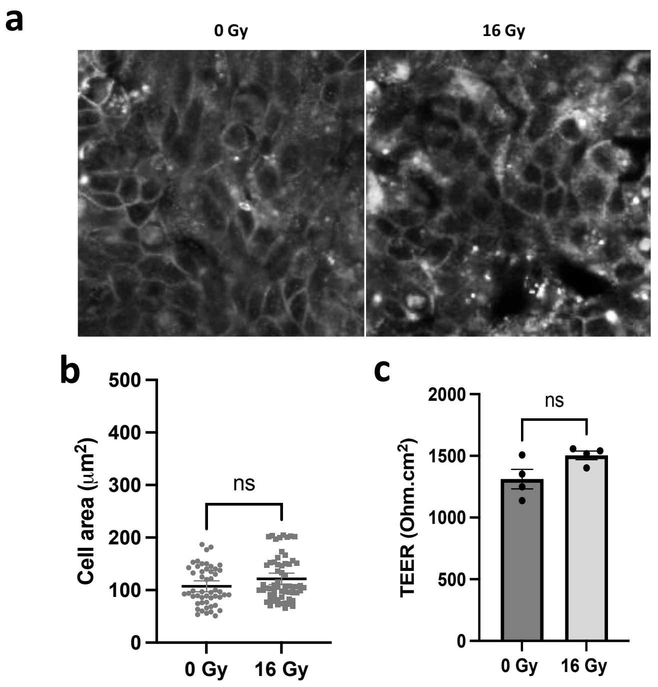

Supplementary Figure S2: (a) Representative Immunofluorescence images of alveolar epithelium in Transwell (E-cad staining). Alveolar cells on transwells do not exhibit hypertrophy in response to 16 Gy radiation (b) Quantification of cell size from E-cad staining images (data plotted from 8 different frames in each condition, n=98 for 0 Gy and n=78 for 16 Gy) (c) Assessment of barrier function shows no difference in the TEER resistance values in response to radiation when the same cells were maintained in Transwell culture.

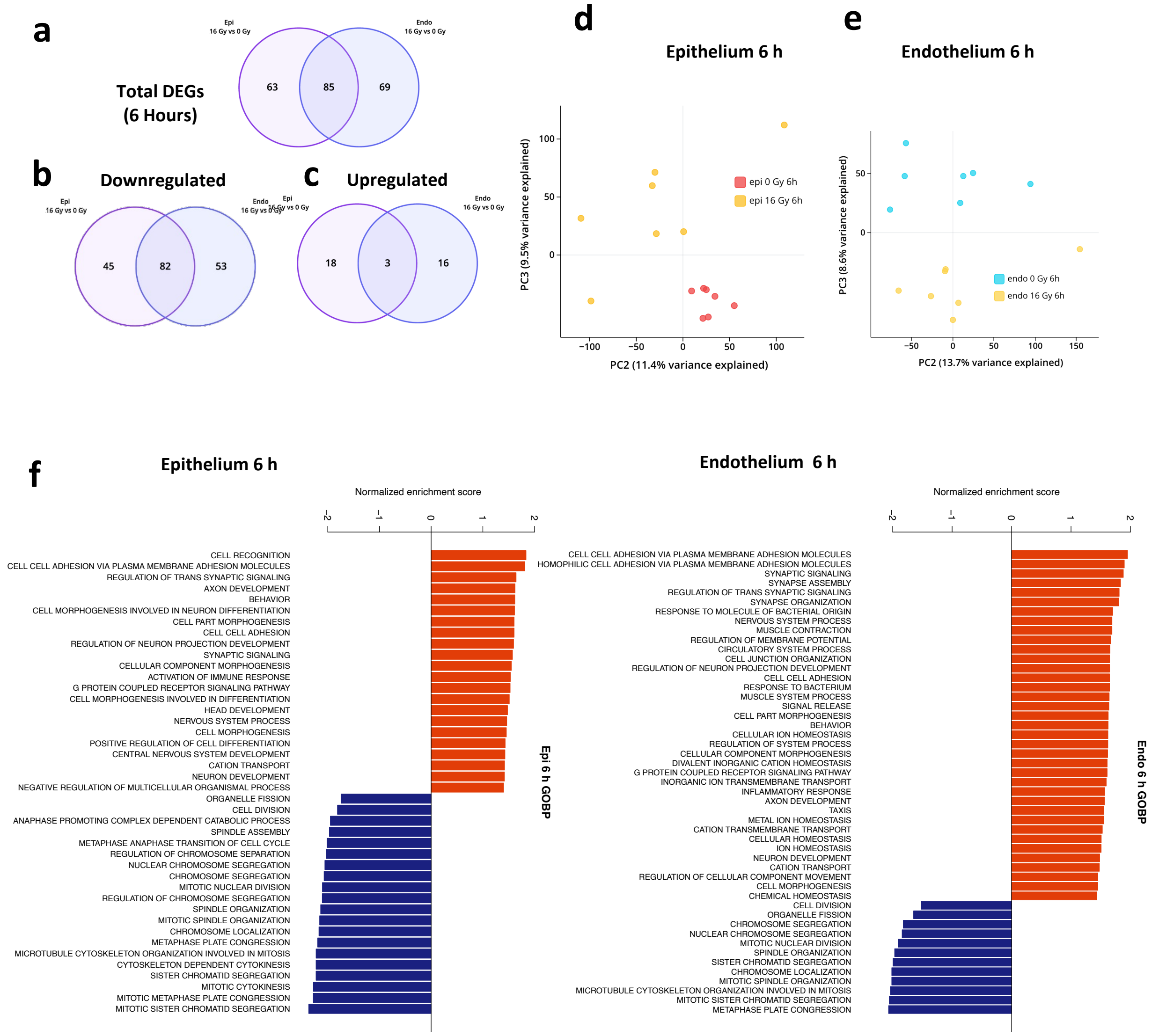

Supplementary Figure S3: Transcriptomic analyses at 6 h post-radiation. (a) Venn diagram showing total differentially expressed genes (DEGs) in response to radiation, 6 h after exposure, (b) Genes downregulated in the epithelium and endothelium, 6 h after radiation (c) Genes upregulated in the epithelium and endothelium, 6 h after radiation. Principal component analysis of the (d) epithelium and (e) endothelium showing the different clusters formed in response to radiation injury. (f) Barplots showing the top significant gene ontology: biological pathways upregulated in response to radiation in the epithelium and endothelium

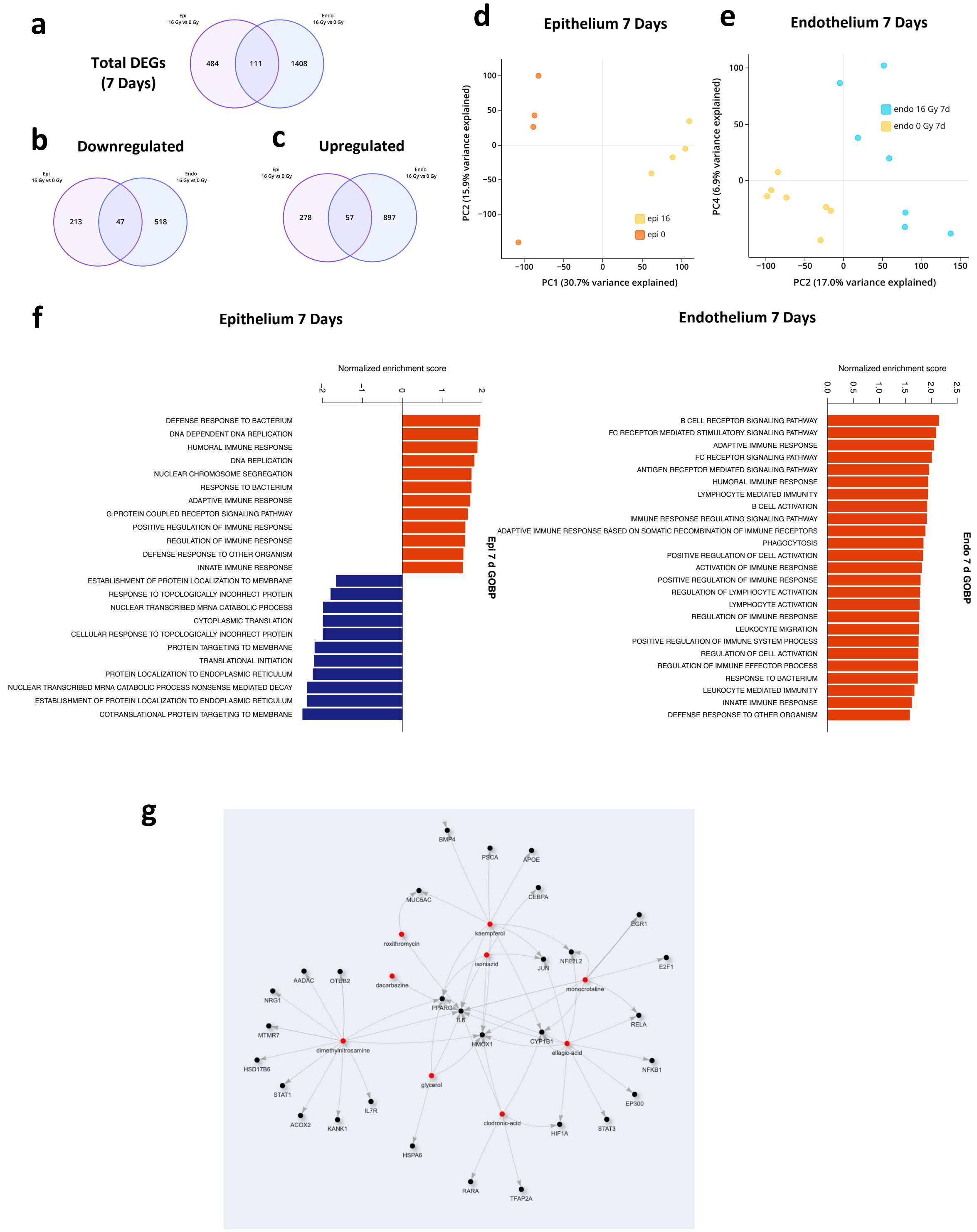

Supplementary Figure S4: Transcriptomic analyses at 7d post-radiation. (a) Venn diagram showing total differentially expressed genes (DEGs) in response to radiation, 7 d after

exposure, (b) Genes downregulated in the epithelium and endothelium, 7 d after radiation (c) Genes upregulated in the epithelium and endothelium, 7 d after radiation Principal component analysis of the (d) epithelium and (e) endothelium showing the different clusters formed in response to radiation injury. (f) Dotplots showing the top significant gene ontology: biological pathways upregulated in response to radiation in the epithelium and endothelium (g) NeMoCAD analysis of the transcriptomic analyses at 7d post radiation showing that HMOX-1 and IL6 are identified as central targets in an agnostic approach.

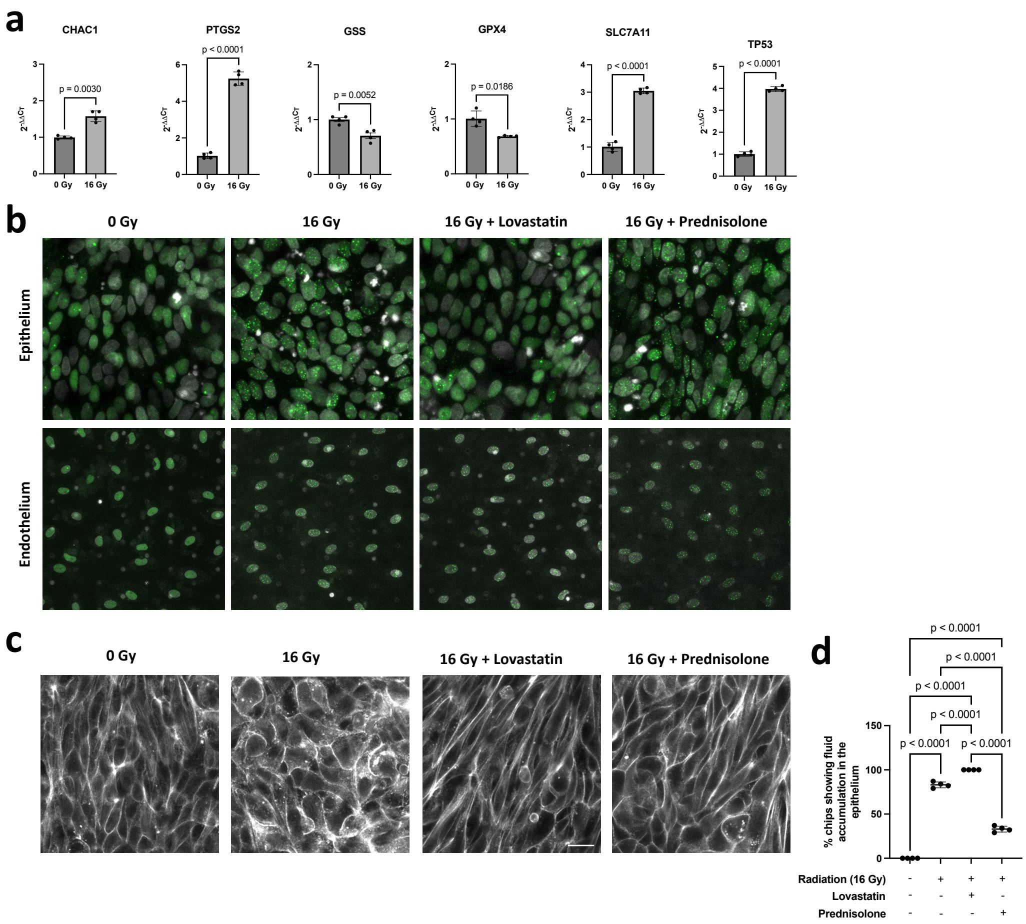

Supplementary Figure S5: (a) RNA expression of ferroptosis-associated markers in response to radiation at 7 d post-exposure. (b) IF imaging showing the effect of lovastatin and prednisolone on formation of 53bp1 nuclear foci (c) cellular hypertrophy observed in the

epithelium (d) % chips exhibiting fluid accumulation in the epithelium within 7 d of radiation exposure.

Table S1. GSEA analysis of Hallmark pathways of the epithelium at 6 h post-radiation (Gene sets with q<0.05)

Gene_Set_Display_Name	P_Value	Adj_P_Value (q)	Size	NES
HALLMARK G2M CHECKPOINT	2.61485210624706E-12	1.30742605312353E-10	193	-2.16543863345178
HALLMARK MITOTIC SPINDLE	5.42146242822788E-11	1.35536560705697E-09	198	-2.0608153506307
HALLMARK E2F TARGETS	5.44163957304786E-09	9.0693992884131E-08	198	-1.92301056393275
HALLMARK ALLOGRAFT REJECTION	0.000184820201308959	0.00231025251636199	154	1.63528706257536
HALLMARK KRAS SIGNALING UP	0.00463142892706744	0.0463142892706744	181	1.43688685545727
HALLMARK INFLAMMATORY RESPONSE	0.00590963682937905	0.0492469735781587	175	1.43131952563214

Table S2. GSEA analysis of Hallmark pathways of the endothelium at 6 h post-radiation (Gene sets with q<0.05)

Gene_Set_Display_Name	P_Value	Adj_P_Value (q)	NES	Size
HALLMARK MITOTIC SPINDLE	2.0692700393136E-09	1.0346350196568E-07	-2.01642700419275	198
HALLMARK G2M CHECKPOINT	1.65638871305701E-08	4.14097178264253E-07	-1.95230423586394	194
HALLMARK INFLAMMATORY RESPONSE	1.13413639635922E-06	1.89022732726536E-05	1.83463697076536	180
HALLMARK E2F TARGETS	3.24386756973101E-05	0.000405483446216376	-1.66243424543345	198
HALLMARK COMPLEMENT	9.52874914903819E-05	0.000914239522592587	1.67463105009828	182
HALLMARK EPITHELIAL MESENCHYMAL TRANSITION	0.00010970874271111	0.000914239522592587	1.65720906232386	196
HALLMARK IL2 STAT5 SIGNALING	0.00046406466897613	0.00331474763554379	1.60022838260003	185
HALLMARK KRAS SIGNALING UP	0.000944194413267172	0.00472097206633586	1.53992807557878	184
HALLMARK MYOGENESIS	0.00090236854617351	0.00472097206633586	1.54377805137751	168
HALLMARK TNFA SIGNALING VIA NFKB	0.000795045361105707	0.00472097206633586	1.54878850553363	197
HALLMARK KRAS SIGNALING DN	0.00221416608007363	0.010064391273062	1.53234048123519	147

Table S3. GSEA analysis of GO: Biological processes of the epithelium at 6 h post-radiation (Top 25 Gene sets with q<0.05)

Gene_Set_Display_Name	P_Value	Adj_P_Value (q)	NES	Size
GOBP MITOTIC SISTER CHROMATID SEGREGATION	4.76658610983329E-15	3.26558814384678E-11	-2.36800368064425	155
GOBP CHROMOSOME SEGREGATION	1.59120799564536E-14	5.45068298908319E-11	-2.07070115416762	295
GOBP SISTER CHROMATID SEGREGATION	4.76890013331094E-14	1.08905782711044E-10	-2.2262600649771	185
GOBP MITOTIC NUCLEAR DIVISION	8.23769345301782E-14	1.41091094616563E-10	-2.10309885582539	274
GOBP CELL DIVISION	2.8788656419052E-13	3.9446217025385E-10	-1.8121894818524	541
GOBP NUCLEAR CHROMOSOME SEGREGATION	1.91446424137984E-12	2.18599908628221E-09	-2.05557674134711	236
GOBP MICROTUBULE CYTOSKELETON ORGANIZATION INVOLVED IN MITOSIS	2.07209849664446E-11	2.02799240007303E-08	-2.22543849324419	138
GOBP SPINDLE ORGANIZATION	3.32511991782888E-11	2.8475495696307E-08	-2.1371996135109	175
GOBP CELL CELL ADHESION	4.84550408516253E-11	3.68850538749428E-08	1.60476154760252	714
GOBP ORGANELLE FISSION	3.19710052191539E-10	2.19033356756423E-07	-1.74256856893587	416
GOBP CELL PART MORPHOGENESIS	8.09036154413169E-10	5.03882426716784E-07	1.6050338267574	608
GOBP MITOTIC SPINDLE ORGANIZATION	1.39763703247708E-09	7.97934275791707E-07	-2.15698104199817	114
GOBP CYTOSKELETON DEPENDENT CYTOKINESIS	2.6349444943983E-09	1.28942890936591E-06	-2.22593611913554	82
GOBP MITOTIC CYTOKINESIS	2.57918907469293E-09	1.28942890936591E-06	-2.2772399603984	67
GOBP CELL MORPHOGENESIS INVOLVED IN NEURON DIFFERENTIATION	2.96409062189461E-09	1.35379899004E-06	1.6132297901035	516
GOBP CELL CELL ADHESION VIA PLASMA MEMBRANE ADHESION MOLECULES	7.4102730679699E-09	3.17298629929136E-06	1.81053860743545	215
GOBP CELLULAR COMPONENT MORPHOGENESIS	1.06878385128826E-08	4.3071989206917E-06	1.55074836516176	672
GOBP AXON DEVELOPMENT	2.36594649666855E-08	9.00505524926457E-06	1.62247104150569	453
GOBP MITOTIC METAPHASE PLATE CONGRESSION	2.51124417655993E-08	9.05501781769058E-06	-2.27965590841784	50
GOBP CELL MORPHOGENESIS	6.88258879531483E-08	2.06519245666711E-05	1.4545788652166	890

GOBP CHROMOSOME LOCALIZATION	6.40987384659501E-08	2.06519245666711E-05	-2.17008853197221	75
GOBP METAPHASE PLATE CONGRESSION	6.93321069965605E-08	2.06519245666711E-05	-2.19480965776565	63
GOBP SYNAPTIC SIGNALING	6.52103451673708E-08	2.06519245666711E-05	1.57290377454781	518
GOBP BEHAVIOR	8.12862903267304E-08	2.32038489595179E-05	1.61858814571334	414
GOBP CELL MORPHOGENESIS INVOLVED IN DIFFERENTIATION	1.70350978901575E-07	4.48874829405651E-05	1.51186193354741	649

Table S4. GSEA analysis of GO: Biological processes of the endothelium at 6 h post-radiation (Top 25 Gene sets with q<0.05)

Gene_Set_Display_Name	P_Value	Adj_P_Value (q)	NES	Size
GOBP SYNAPTIC SIGNALING	1.58289307024412E-13	1.08364859588912E-09	1.87896303203607	542
GOBP NERVOUS SYSTEM PROCESS	2.656175652921E-10	9.09208925994859E-07	1.68047911964966	699
GOBP CELL CELL ADHESION VIA PLASMA MEMBRANE ADHESION MOLECULES	8.87817464882272E-10	2.02599945486135E-06	1.95395744207122	216
GOBP CELL CELL ADHESION	1.34361114736924E-09	2.29959047872245E-06	1.64376308820884	721
GOBP MITOTIC NUCLEAR DIVISION	2.22083566524819E-09	3.04076819285783E-06	-1.8840575456738	276
GOBP SYNAPSE ORGANIZATION	4.60790851643189E-09	5.25762361724879E-06	1.80294086639882	348
GOBP REGULATION OF TRANS SYNAPTIC SIGNALING	5.46974660182869E-09	5.34941217658846E-06	1.80591834355575	335
GOBP CELLULAR COMPONENT MORPHOGENESIS	9.22323083039388E-09	7.01580425165295E-06	1.60467866986679	671
GOBP SISTER CHROMATID SEGREGATION	8.70931770532033E-09	7.01580425165295E-06	-1.99792255115774	185
GOBP MITOTIC SISTER CHROMATID SEGREGATION	1.14010517521149E-08	7.80516002949786E-06	-2.05387715086106	155
GOBP MICROTUBULE CYTOSKELETON ORGANIZATION INVOLVED IN MITOSIS	1.3430099807388E-08	8.35840575285259E-06	-2.05491133743229	137
GOBP CELL JUNCTION ORGANIZATION	2.04626562590122E-08	1.10990695361506E-05	1.64778526741734	605
GOBP SPINDLE ORGANIZATION	2.10762348772945E-08	1.10990695361506E-05	-1.97442842458931	175
GOBP CHROMOSOME SEGREGATION	3.39089863383225E-08	1.59597908114183E-05	-1.80117466985391	296
GOBP NUCLEAR CHROMOSOME SEGREGATION	3.49688668085413E-08	1.59597908114183E-05	-1.846998937382	237

GOBP CELL PART MORPHOGENESIS	4.58084779739676E-08	1.87421793252085E-05	1.62304013747548	608
GOBP G PROTEIN COUPLED RECEPTOR SIGNALING PATHWAY	4.92782979628619E-08	1.87421793252085E-05	1.60667431389392	604
GOBP INORGANIC ION TRANSMEMBRANE TRANSPORT	4.87154747627785E-08	1.87421793252085E-05	1.58800249325027	636
GOBP CIRCULATORY SYSTEM PROCESS	1.97617665516606E-07	7.12047651645623E-05	1.65752453984743	471
GOBP ORGANELLE FISSION	2.27951947920592E-07	7.80279517732186E-05	-1.64417256823422	420
GOBP MITOTIC SPINDLE ORGANIZATION	2.40214113195023E-07	7.83098009015774E-05	-2.01304482752988	114
GOBP CELLULAR ION HOMEOSTASIS	2.82513829569544E-07	8.79131671469591E-05	1.61979688069021	516
GOBP RESPONSE TO BACTERIUM	4.35585316699037E-07	0.00012965291644007	1.64571079679719	442
GOBP NEURON DEVELOPMENT	5.79272250492929E-07	0.000165237409453108	1.48457692649649	967
GOBP INFLAMMATORY RESPONSE	7.19532600240781E-07	0.000189458468509553	1.56340425596444	554

Table S5. GSEA analysis of Hallmark pathways of the epithelium at 7 d post-radiation (Gene sets with q<0.05)

Gene_Set_Display_Name	P_Value	Adj_P_Value (q)	NES	Size
HALLMARK E2F TARGETS	2.40268955252172E-13	1.20134477626086E-11	2.16643233047431	197
HALLMARK G2M CHECKPOINT	7.62717348822171E-09	1.90679337205543E-07	1.97276575708238	194
HALLMARK INTERFERON GAMMA RESPONSE	1.66692649477616E-07	2.77821082462693E-06	1.88064244883232	189
HALLMARK INTERFERON ALPHA RESPONSE	3.77727437666473E-07	4.72159297083091E-06	2.01068299800561	95
HALLMARK P53 PATHWAY	0.00291428369543438	0.0291428369543438	-1.43031457250602	193
HALLMARK ALLOGRAFT REJECTION	0.00718784804106784	0.0513417717219132	1.49765693114821	159
HALLMARK INFLAMMATORY RESPONSE	0.00688101557978458	0.0513417717219132	1.4618037691579	177

Table S6. GSEA analysis of Hallmark pathways of the endothelium at 7 d post-radiation (Gene sets with q<0.05)

Gene_Set_Display_Name	P_Value	Adj_P_Value (q)	NES	Size

**HALLMARK ALLOGRAFT REJECTION**	5.9837291078719E-15	2.5131662253062E-13	1.81279024120861	183
**HALLMARK INFLAMMATORY RESPONSE**	7.17247805116516E-11	1.50622039074468E-09	1.69829541333824	191
**HALLMARK KRAS SIGNALING UP**	9.33580323222341E-07	1.30701245251128E-05	1.55517120692485	191
**HALLMARK INTERFERON GAMMA RESPONSE**	2.15180578371936E-06	2.25939607290533E-05	1.52588900980021	198
**HALLMARK MYC TARGETS V2**	2.82127864183521E-06	2.36987405914158E-05	-2.18141335601015	58
**HALLMARK COMPLEMENT**	1.37129116867258E-05	9.59903818070807E-05	1.48757601640123	192
**HALLMARK TNFA SIGNALING VIA NFKB**	3.28667586813698E-05	0.000197200552088219	1.467430301239	199
**HALLMARK IL2 STAT5 SIGNALING**	4.69590503057674E-05	0.000246535014105279	1.45749369128056	193
**HALLMARK IL6 JAK STAT3 SIGNALING**	0.000114297423011294	0.000533387974052705	1.58709761334046	85
**HALLMARK EPITHELIAL MESENCHYMAL TRANSITION**	0.0135394528662318	0.0498657020381736	1.2823952359935	198

Table S7. GSEA analysis of GO: Biological processes of the epithelium at 7 d post-radiation (Top 25 Gene sets with q<0.05)

Gene_Set_Display_Name	P_Value	Adj_P_Value (q)	NES	Size
**GOBP COTRANSLATIONAL PROTEIN TARGETING TO MEMBRANE**	1.03154158314717E-11	4.24974768524057E-08	-2.3934307401169	104
**GOBP ESTABLISHMENT OF PROTEIN LOCALIZATION TO ENDOPLASMIC RETICULUM**	2.26006175833246E-11	4.24974768524057E-08	-2.32794190265398	117
**GOBP PROTEIN TARGETING TO MEMBRANE**	1.86203911431052E-11	4.24974768524057E-08	-2.13975957997319	192
**GOBP TRANSLATIONAL INITIATION**	2.46362184651627E-11	4.24974768524057E-08	-2.13641669698471	185
**GOBP NUCLEAR TRANSCRIBED MRNA CATABOLIC PROCESS NONSENSE MEDIATED DECAY**	3.61153541238436E-11	4.98391886909041E-08	-2.33357120746739	120
**GOBP PROTEIN LOCALIZATION TO ENDOPLASMIC RETICULUM**	3.620909714052E-10	4.1640461711598E-07	-2.1991190163559	142
**GOBP RESPONSE TO BACTERIUM**	1.85613245033454E-08	1.82961627247261E-05	1.71295288314043	458
**GOBP G PROTEIN COUPLED RECEPTOR SIGNALING PATHWAY**	3.13692882415861E-08	2.7056011108368E-05	1.62139690774893	623

GOBP DEFENSE RESPONSE TO BACTERIUM	4.22205354715191E-08	3.23690771948313E-05	1.94122814633922	159
GOBP NUCLEAR TRANSCRIBED MRNA CATABOLIC PROCESS	5.68359425531994E-08	3.92168003617076E-05	-1.92939229505672	201
GOBP DEFENSE RESPONSE TO OTHER ORGANISM	6.64048039291652E-08	4.16539224646582E-05	1.51885496721504	842
GOBP DNA REPLICATION	1.52362941930173E-07	8.14522191474761E-05	1.80586445055213	264
GOBP REGULATION OF IMMUNE RESPONSE	1.53460702741622E-07	8.14522191474761E-05	1.56428253643073	708
GOBP CELLULAR RESPONSE TO TOPOLOGICALLY INCORRECT PROTEIN	2.2033841949199E-07	0.000108595363892481	-1.93413235206918	163
GOBP DNA DEPENDENT DNA REPLICATION	4.02024667083945E-07	0.000184931346858615	1.89752178666229	148
GOBP INNATE IMMUNE RESPONSE	8.6958370471779E-07	0.000375007972659547	1.5086530216135	692
GOBP HUMORAL IMMUNE RESPONSE	1.00705781692806E-06	0.000408746996282564	1.87235110271223	169
GOBP ADAPTIVE IMMUNE RESPONSE	1.0694556588104E-06	0.000409958002543986	1.68990754376123	326
GOBP POSITIVE REGULATION OF IMMUNE RESPONSE	1.40538498766029E-06	0.000510376653413474	1.56461715648231	522
GOBP CHROMOSOME SEGREGATION	1.9625677544075E-06	0.000662896086759687	1.67161471322911	297
GOBP ESTABLISHMENT OF PROTEIN LOCALIZATION TO MEMBRANE	2.01750982926861E-06	0.000662896086759687	-1.61507672919611	327
GOBP RESPONSE TO TOPOLOGICALLY INCORRECT PROTEIN	2.46638971031487E-06	0.000773549500053299	-1.74111455379986	198
GOBP SISTER CHROMATID SEGREGATION	3.90325108273863E-06	0.00117097532482159	1.77705249279709	186
GOBP NUCLEAR CHROMOSOME SEGREGATION	4.81955502123565E-06	0.00138562206860525	1.72146737073596	238
GOBP CYTOPLASMIC TRANSLATION	5.51703453257639E-06	0.00152270153099108	-1.91008287090094	99

Table S8. GSEA analysis of GO: Biological processes of the endothelium at 7 d post-radiation (Top 25 Gene sets with q<0.05)

Gene_Set_Display_Name	P_Value	Adj_P_Value (q)	NES	Size
GOBP ADAPTIVE IMMUNE RESPONSE	8.32286560691616E-76	5.72363467787624E-72	2.0549742196439	531
GOBP POSITIVE REGULATION OF IMMUNE SYSTEM PROCESS	9.72623527292456E-52	3.34436599859511E-48	1.746642879911	910

**GOBP REGULATION OF IMMUNE RESPONSE**	1.18188581649144E-49	2.70927625333721E-46	1.75395501352127	861
**GOBP IMMUNE RESPONSE REGULATING SIGNALING PATHWAY**	1.29321702960316E-44	2.22336337814524E-41	1.90822415307961	454
**GOBP POSITIVE REGULATION OF IMMUNE RESPONSE**	2.75004608532759E-42	3.78241338575956E-39	1.78782719227402	641
**GOBP LYMPHOCYTE ACTIVATION**	1.07251823520817E-40	1.22928465058776E-37	1.76907851760509	659
**GOBP ACTIVATION OF IMMUNE RESPONSE**	1.82615167314137E-35	1.79406357945617E-32	1.81549812241543	484
**GOBP LEUKOCYTE MEDIATED IMMUNITY**	9.184575852596E-34	7.89529101728784E-31	1.66595067791409	776
**GOBP REGULATION OF CELL ACTIVATION**	9.48415292111218E-33	7.24694662649872E-30	1.74979969339755	551
**GOBP ANTIGEN RECEPTOR MEDIATED SIGNALING PATHWAY**	4.76707532233133E-32	3.27831769916725E-29	1.95071627635422	279
**GOBP LYMPHOCYTE MEDIATED IMMUNITY**	5.77469832251156E-31	3.61023639671927E-28	1.92703239085621	287
**GOBP RESPONSE TO BACTERIUM**	9.40171492159961E-31	5.38796612632005E-28	1.73886201060686	549
**GOBP REGULATION OF LYMPHOCYTE ACTIVATION**	4.14607413118933E-30	2.19327321539916E-27	1.78023145363531	444
**GOBP FC RECEPTOR SIGNALING PATHWAY**	1.37845269126215E-29	6.77115654129271E-27	2.00753702645478	221
**GOBP DEFENSE RESPONSE TO OTHER ORGANISM**	1.89398235332678E-29	8.68327776255218E-27	1.57629616527519	965
**GOBP INNATE IMMUNE RESPONSE**	3.6808327884041E-29	1.58206794286594E-26	1.61732524750558	802
**GOBP ADAPTIVE IMMUNE RESPONSE BASED ON SOMATIC RECOMBINATION OF IMMUNE RECEPTORS BUILT FROM IMMUNOGLOBULIN SUPERFAMILY DOMAINS**	1.0938878203098E-28	4.42509796486499E-26	1.88139625824775	297
**GOBP HUMORAL IMMUNE RESPONSE**	3.8058568118847E-28	1.45404873862951E-25	1.9307121205075	247
**GOBP POSITIVE REGULATION OF CELL ACTIVATION**	1.22787353681929E-27	4.44425595405591E-25	1.82954287119586	351
**GOBP B CELL ACTIVATION**	5.71037515915391E-27	1.87001190330959E-24	1.91384925109849	264
**GOBP LEUKOCYTE MIGRATION**	5.71037515915391E-27	1.87001190330959E-24	1.75860228370738	441
**GOBP PHAGOCYTOSIS**	7.79442162174984E-27	2.43646534058062E-24	1.84280457350115	318
**GOBP FC RECEPTOR MEDIATED STIMULATORY SIGNALING PATHWAY**	2.06161508736514E-24	6.16422911122176E-22	2.10754981587759	130

**GOBP B CELL RECEPTOR SIGNALING PATHWAY**	5.96907220782316E-24	1.71038789888333E-21	2.15733966412002	100
**GOBP REGULATION OF IMMUNE EFFECTOR PROCESS**	7.39809791700813E-24	2.0350687750106E-21	1.7377963270103	398

Table S9. List of Thermo Fisher Taqman probes

Gene symbol	Cat. no.	Assay ID
HPRT1	4331182	Hs02800695_m1
SNAI1	4331182	Hs00195591_m1
B2M	4351370	Hs00187842_m1
AQP5	4331182	Hs00387048_m1
SFTPC	4331182	Hs00161628_m1
SFTPB	4331182	Hs00167036_m1
ACTA2	4331182	Hs00909449_m1
PDPN	4331182	Hs00366766_m1
ICAM1	4331182	Hs00164932_m1
SELE	4331182	Hs00950401_m1
ATM	4331182	Hs00175892_m1
GPX4	4331182	Hs00989766_g1
H2AFX	4331182	Hs00266783_s1
DDIT3	4331182	Hs00358796_g1
CHAC1	4331182	Hs00225520_m1
PTGS2	4331182	Hs00153133_m1
HMOX1	4331182	Hs01110250_m1
P21	4331182	Hs01371942_m1
TP53	4331182	Hs01034249_m1

BAX	4331182	Hs00180269_m1
BCL2	4331182	Hs04986394_s1
GSS	4331182	Hs00609285_m1
SLC7A11	4351372	Hs00921933_m1

Table S10. List of antibodies used in immunostaining.

**Antibody**	**Company**	**Cat. No.**	**Dilution**
Anti-E Cadherin antibody	Abcam	ab1416	1:100
	Novus Biologicals	FAB748R-025	1:100
Anti-ZO1 tight junction protein antibody - C-terminal	Abcam	ab190085	1:50
Anti-CD31 antibody [JC/70A]	Abcam	ab9498	1:100
Anti-53BP1 antibody	Abcam	ab36823	1:100
	Novus Biologicals	NB100-305AF594	1:100
VE-Cadherin	Cell Signaling Technologies	2500S	1:50
	Novus Biologicals	FAB9381X	1:100
anti-mouse IgG-488	Invitrogen	A-1100	1: 1000
anti-mouse IgG-647	Invitrogen	A-21235	1: 1000
anti-rabbit IgG-488	Invitrogen	A-11034	1: 1000
anti-rabbit IgG-647	Invitrogen	A-32733	1: 1000
