# Supplementary material for: A human lung alveolus-on-a-chip model of acute radiation-induced lung injury
Source: Nat Commun. 2023 Oct 16;14:6506. doi: 10.1038/s41467-023-42171-z (PMC10579267; doi:10.1038/s41467-023-42171-z)
Supplement: Supplementary file 3 — Reporting Summary [file 41467_2023_42171_MOESM3_ESM.pdf]

nature portfolio

Corresponding author(s): Donald E. Ingber

Last updated by author(s): Aug 26, 2023

Reporting Summary

Nature Portfolio wishes to improve the reproducibility of the work that we publish. This form provides structure for consistency and transparency in reporting. For further information on Nature Portfolio policies, see our Editorial Policies and the Editorial Policy Checklist.

Statistics

For all statistical analyses, confirm that the following items are present in the figure legend, table legend, main text, or Methods section.

n/a	Confirmed	
☐	☒	The exact sample size (*n*) for each experimental group/condition, given as a discrete number and unit of measurement
☐	☒	A statement on whether measurements were taken from distinct samples or whether the same sample was measured repeatedly
☐	☒	The statistical test(s) used AND whether they are one- or two-sided<br>*Only common tests should be described solely by name; describe more complex techniques in the Methods section.*
☒	☐	A description of all covariates tested
☐	☒	A description of any assumptions or corrections, such as tests of normality and adjustment for multiple comparisons
☐	☒	A full description of the statistical parameters including central tendency (e.g. means) or other basic estimates (e.g. regression coefficient) AND variation (e.g. standard deviation) or associated estimates of uncertainty (e.g. confidence intervals)
☐	☒	For null hypothesis testing, the test statistic (e.g. *F*, *t*, *r*) with confidence intervals, effect sizes, degrees of freedom and *P* value noted<br>*Give P values as exact values whenever suitable.*
☒	☐	For Bayesian analysis, information on the choice of priors and Markov chain Monte Carlo settings
☒	☐	For hierarchical and complex designs, identification of the appropriate level for tests and full reporting of outcomes
☒	☐	Estimates of effect sizes (e.g. Cohen's *d*, Pearson's *r*), indicating how they were calculated

Our web collection on statistics for biologists contains articles on many of the points above.

Software and code

Policy information about availability of computer code

Data collection	Images were taken with the built-in software of a confocal laser-scanning microscope (ZEN imaging software, v3.7 and SP5 X MP DMI-6000, Germany). Fiji (Image J, V2.1.0) was used to analyze images. Cytokine and chemokine concentrations were evaluated using a Bio-Plex 3D suspension array system and analyzed for standard curve fitting and concentration calculations with Bio-Plex Manager software (Bio-Rad, v 6.0). qPCR experiments were conducted using ExpressionSuite software v1.3 (Applied Biosystems).
Data analysis	GraphPad Prism v9.4.0 for plotting, graphing and statistical analyses. Pluto (www.pluto.bio) for creating plots and analyses of bulk RNA-sequencing data. Package DESeq2 version 1.28.1, Package ggplot2 version 3.3.2, Package EnhancedVolcano version 1.6.0, Package pheatmap version 1.0.12, Short Time-series Expression Miner (STEM) version 1.3.13 , Trimmomatic v.0.36, STAR aligner v.2.5.2b, Subread package v.1.5.2 , DAVID version 6.8, Enrichplot R package version 1.14.2, Microsoft Excel v16.53,

For manuscripts utilizing custom algorithms or software that are central to the research but not yet described in published literature, software must be made available to editors and reviewers. We strongly encourage code deposition in a community repository (e.g. GitHub). See the Nature Portfolio guidelines for submitting code & software for further information.

Data

Policy information about availability of data

All manuscripts must include a data availability statement. This statement should provide the following information, where applicable:

- Accession codes, unique identifiers, or web links for publicly available datasets
- A description of any restrictions on data availability
- For clinical datasets or third party data, please ensure that the statement adheres to our policy

All bulk RNA sequencing data have been uploaded on the Gene Expression Omnibus (GEO) database and made publicly available with accession numbers GSE242706 and GSE242840. Additional data are available in the Supplementary information. The data were analyzed using available data packages mentioned in the manuscript and no new or custom codes were created to analyze the data. There are no restrictions on data availability and raw data has been made available.

Research involving human participants, their data, or biological material

Policy information about studies with human participants or human data. See also policy information about sex, gender (identity/presentation), and sexual orientation and race, ethnicity and racism.

Reporting on sex and gender	NA
Reporting on race, ethnicity, or other socially relevant groupings	NA
Population characteristics	NA
Recruitment	NA
Ethics oversight	NA

Note that full information on the approval of the study protocol must also be provided in the manuscript.

Field-specific reporting

Please select the one below that is the best fit for your research. If you are not sure, read the appropriate sections before making your selection.

☒ Life sciences ☐ Behavioural & social sciences ☐ Ecological, evolutionary & environmental sciences

For a reference copy of the document with all sections, see nature.com/documents/nr-reporting-summary-flat.pdf

Life sciences study design

All studies must disclose on these points even when the disclosure is negative.

Sample size	For the human-Alveolus-chip studies, 4 to 6 chips were used based on prior experience with organ chip variations, similar to what is described for similar experiments in published articles (Bai et al., Nature Communictaions, 2022, Si et al., Nature Biomedical Engineering, 2021; Benam et al,, Nature Methods, 2016). We did not use any statistical methods to predetermine sample size. The sample size for all other experiments was chosen to include at least 3 biologically independent experiments. The exact numbers are described in each figure legend.
Data exclusions	No data exclusions were made
Replication	Each experiment with a specific set of donors has been repeated at least at 3 different times, under the same conditions. In order to verify the reproducibility of the experimental findings, all cell studies for specific donors were performed at least in triplicate. All human alveolus chip studies were performed at least in duplicate. All the attempts were successful except for 5-8% of the chips that failed during the culture process (eg. they do not successfully form an air-liquid interface or have different vascular flow than expected). Those chips were not used for radiation injury or drug testing, and no data were generated using them.
Randomization	The organ chips were assigned into random positions on the Zoe culture equipment in these studies. Alveolus chips were randomly assigned to the Control (no radiation) or radiated (exposed to radiation) groups
Blinding	Data collection and analysis for cytokines and chemokines in human alveolus chips, gene levels detected by RT-qPCR, and imaging were run blinded, with one researcher responsible for collecting samples and another researcher for running assays. Thus, they did not know sample identity until data was analyzed. Blinding was not applicable to other experiments because they were quantitative and controlled. RNA-seq was performed by a third party at Azenta Life Sciences and the samples were numbered in a way to mask identity

Reporting for specific materials, systems and methods

We require information from authors about some types of materials, experimental systems and methods used in many studies. Here, indicate whether each material, system or method listed is relevant to your study. If you are not sure if a list item applies to your research, read the appropriate section before selecting a response.

Materials & experimental systems

n/a	Involved in the study
☐	☒ Antibodies
☐	☒ Eukaryotic cell lines
☒	☐ Palaeontology and archaeology
☒	☐ Animals and other organisms
☒	☐ Clinical data
☒	☐ Dual use research of concern
☒	☐ Plants

Methods

n/a	Involved in the study
☒	☐ ChIP-seq
☒	☐ Flow cytometry
☒	☐ MRI-based neuroimaging

Antibodies

Antibodies used

All antibody data are included in the supplementary information of the manuscript.
Anti-E Cadherin antibody Abcam ab1416 (dilution 1: 100), Novus Biologicals FAB748R-025 (dilution 1: 100)
Anti-ZO1 tight junction protein antibody - C-terminal Abcam ab190085 (dilution 1: 50)
Anti-CD31 antibody [JC/70A] Abcam ab9498 (dilution 1: 100)
Anti-53BP1 antibody Abcam ab36823 (dilution 1: 100), Novus Biologicals NB100-305AF594 (dilution 1: 100)
VE-Cadherin Cell Signaling Technologies 2500S (dilution 1: 50) , Novus Biologicals FAB9381X (dilution 1: 100)
anti-mouse IgG-488 (Invitrogen, A-11001, dilution 1: 1000), anti-mouse IgG-647 (Invitrogen, A-21235, dilution 1:1000), anti-rabbit IgG-488 (Invitrogen, A-11034, dilution 1: 1000), anti-rabbit IgG-647 (Invitrogen, A-32733, dilution 1: 1000)

Validation

All antibody validation data are available on the manufacturer's website. We verified information provided in the corresponding Data Sheets provided by the manufacturers and confirm that they have been validated for immunofluorescence or flow cytometry for humans. We also confirmed that these antibodies had been used in previous publications.

Eukaryotic cell lines

Policy information about cell lines and Sex and Gender in Research

Cell line source(s)

Primary human lung microvascular endothelial cells (Lonza, CC-2527, P3), primary human lung alveolar epithelial cells (Cell Biologics, P3, H-6053, Lot F101517Y72, F120318Y58, 120718Y31), Peripheral blood mononuclear cells (PBMCs) (Stemcell Technologies, 70025.1)

Authentication

Primary human lung microvascular endothelial cells were used within passage 5. Primary human lung alveolar epithelial cells were used directly upon thawing without expanding. PBMCs were also used directly after thawing without expansion. All cells showed expected cell morphology, growth behaviour, and cell type-specific responses. No further authentication was performed.

Mycoplasma contamination

All cells were examined and found to be negative for mycoplasma contamination.

Commonly misidentified lines
(See ICLAC register)

No commonly misidentified cell lines were used in this work.
